# Impact of diabetic kidney disease on post-operative complications after primary elective total hip arthroplasty: a nationwide database analysis

**DOI:** 10.1186/s12891-024-07653-1

**Published:** 2024-07-16

**Authors:** An-dong Zhou, Jie Ding, Qi Zhou, Qin-feng Yang, Xiang Cai, Yi Shi, Hui-yu Zou, Meng-yin Cai

**Affiliations:** 1 Department of Endocrinology & Metabolism, The Third Affiliated Hospital of Sun Yat-Sen University, Guangzhou, 510630 Guangdong China; 2 Guangdong Provincial Key Laboratory of Diabetology, The Third Affiliated Hospital of Sun Yat-Sen University, Guangzhou, 510630 Guangdong China; 3 Division of Orthopaedic Surgery, Department of Orthopaedics, Nanfang Hospital, Southern Medical University, Guangzhou, 510515 Guangdong China; 4 Guangzhou Municipal Key Laboratory of Mechanistic and Translational Obesity Research, The Third Affiliated Hospital of Sun Yat-Sen University, Guangzhou, 510630 Guangdong China; 5 Medical Center for Comprehensive Weight Control, The Third Affiliated Hospital of Sun Yat-Sen University, Guangzhou, 510630 Guangdong China; 6 Huiqiao Medical Center, Nanfang Hospital, Southern Medical University, 1838 Guangzhou Avenue, Guangzhou, 510515 Guangdong China

**Keywords:** Total hip arthroplasty, Diabetic kidney disease, Acute kidney injury, Nationwide inpatient sample, Outcomes, Comorbidity

## Abstract

**Background:**

The high prevalence of diabetic kidney disease (DKD) in the United States necessitates further investigation into its impact on complications associated with total hip arthroplasty (THA). This study utilizes a large nationwide database to explore risk factors in DKD cases undergoing THA.

**Methods:**

This research utilized a case–control design, leveraging data from the national inpatient sample for the years 2016 to 2019. Employing propensity score matching (PSM), patients diagnosed with DKD were paired on a 1:1 basis with individuals free of DKD, ensuring equivalent age, sex, race, Elixhauser Comorbidity Index (ECI), and insurance coverage. Subsequently, comparisons were drawn between these PSM-matched cohorts, examining their characteristics and the incidence of post-THA complications. Multivariate logistic regression analysis was then employed to evaluate the risk of early complications after surgery.

**Results:**

DKD's prevalence in the THA cohort was 2.38%. A 7-year age gap separated DKD and non-DKD patients (74 vs. 67 years, *P* < 0.0001). Additionally, individuals aged above 75 exhibited a substantial 22.58% increase in DKD risk (49.16% vs. 26.58%, *P* < 0.0001). Notably, linear regression analysis yielded a significant association between DKD and postoperative acute kidney injury (AKI), with DKD patients demonstrating 2.274-fold greater odds of AKI in contrast with non-DKD individuals (95% CI: 2.091–2.473).

**Conclusions:**

This study demonstrates that DKD is a significant risk factor for AKI in patients undergoing total hip arthroplasty. Optimizing preoperative kidney function through appropriate interventions might decrease the risk of poor prognosis in this population. More prospective research is warranted to investigate the potential of targeted kidney function improvement strategies in reducing AKI rates after THA. The findings of this study hold promise for enhancing preoperative counseling by surgeons, enabling them to provide DKD patients undergoing THA with more precise information regarding the risks associated with their condition.

## Background

Total hip arthroplasty (THA) has emerged as a cornerstone of orthopedic intervention, demonstrably enhancing functional outcomes and quality of life in populations suffering from advanced osteoarthritis [1, 2]. It is now understood that the growing geriatric demographic and continuous refinement of surgical techniques have been driving the escalating demand for THA [3]. Despite proactive measures, inherent vulnerabilities within the perioperative period can manifest as complications, generating a substantial economic burden on healthcare systems. This burden was estimated to be as high as US$15 billion annually in the US alone and leads to subpar clinical results, including fatalities [3]. Consequently, recent endeavors have prioritized individualized therapy to achieve improved clinical outcomes and cost benefits [4].

The presence of diabetes mellitus (DM) reportedly contributes to alterations in bone and cartilage metabolism, a phenomenon well-documented in the geriatric population [5]. There is an increasing consensus suggesting that diabetes is related to higher susceptibility to fractures and secondary osteoporosis [6, 7]. Therefore, DM is associated with an increased risk of undergoing orthopedic surgery, particularly THA. Furthermore, diabetes has been associated with postoperative complications in THA, including prosthetic joint infection, persistent pain, and increased hospital costs, particularly when diabetes is poorly controlled [8]. Previous investigations employing various methodologies have documented elevated complication rates and suboptimal outcomes, including increased implant loosening and dislocation, in patients with CKD undergoing total hip arthroplasty [9]. Diabetic kidney disease (DKD) is widely acknowledged as a chronic complication of diabetes and one of the leading causes of CKD [10]. However, to the best of our knowledge, the intricate interplay between DKD, postoperative complications, and healthcare expenditure in THA patients has hitherto remained unexplored in large-scale analyses.

Considering the potential impact of DKD on perioperative outcomes and clinical trajectories in THA patients, this study aimed to leverage the National Inpatient Sample (NIS) database to investigate: (1) in-hospital mortality in patients with DKD undergoing THA; (2) demographic profiles of THA recipients; (3) morbidity burden associated with surgical and medical complications; and (4) healthcare resource utilization, as measured by length of stay (LOS) and overall hospitalization expenses, in populations with DKD undergoing THA.

## Methods

### Data resource

This study utilized data from the National Inpatient Sample (NIS), a nationally representative, all-payer inpatient care database maintained by the Healthcare Cost and Utilization Project (HCUP) within the Agency for Healthcare Research and Quality (AHRQ). The NIS comprises a stratified sample of approximately 20% of annual hospitalizations from over 1,000 hospitals across the United States, offering robust generalizability to the national population [11].

### Data collection

The information on patients undergoing THA was collected from the NIS database from 2016 to 2019. The data collected includes diagnoses, demographics, and procedures defined by the International Classification of Disease, 10th edition (ICD-10) diagnosis and procedure codes, LOS, hospital costs, and insurance type. ICD-10-CM procedure codes were used to identify THA patients (*n* = 464,671). Patients receiving THA were then divided into two groups, those with DKD and those without DKD (ICD-10-CM codes were E0821/E0822/E0829/E0921/E0922/E0929/E1021/E1022/E1029/E1121/E1122/E1129/E1321/E1322/E1329). Simultaneously, patients undergoing THA were examined for perioperative complications using the ICD-10-CM diagnosis code.

The term “any complication” was used when at least one surgical or medical complication was documented. Medical complications included acute postoperative anemia, thrombocytopenia, intubation, acute renal failure (ARF), acute myocardial infarction (AMI), pneumonia, pulmonary embolism (PE), stroke, postoperative delirium (PD), urinary tract infection (UTI), deep vein thrombosis/thrombophlebitis (DVT), sepsis, postoperative shock, and blood transfusion. Surgical complications included wound infection, wound dehiscence, hematoma, injury to the peripheral nerve of the lower limb, periprosthetic joint infection, mechanical loosening of a prosthetic joint, dislocation of a prosthetic joint, peri-prosthetic fracture around prosthetic and other prosthetic-related complication [12, 13]. Length of stay was computed as the elapsed time between hospital admission and discharge, with cost data concurrently extracted from the healthcare database to assess the economic burden of the hospitalization episode.

**Fig.1:**
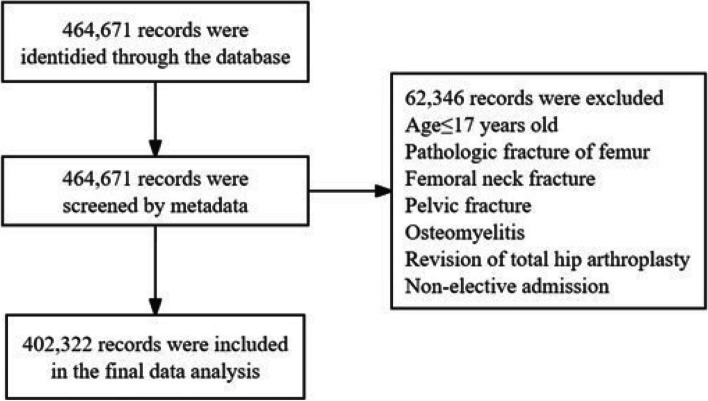
Flow diagram of complications and hospitalization costs in patients with DKD following total hip arthroplasty

Individuals with pre-existing conditions or specific characteristics potentially influencing THA outcomes were excluded, including patients with pathologic fracture of the neck of femur (M84451/M84452/M84551/M84552/M84553/M80851), femoral neck fracture (S700/S703/S704), traumatic pelvic fractures (S321-S326/S328), osteomyelitis(M86051/ M86052/ M86059/ M86151/ M86152/ M86159/ M86251/ M86252/ M86259/ M86351/ M86352/ M86359/ M86451/ M86452/ M86459/ M86551/ M86552/ M86559/ M86651/ M86652/ M86659/ M868X5/ M869), revision THA (0sw9/0swa/0swb/0swe/0swr/0sws), age less than 18 years, and non-elective admission (*n* = 62,349) (Fig. 1). A total of 9583 DKD patients who underwent THA from 2016 to 2019 were detected (Table 1).

**Table 1:** Demographics of total hip arthroplasty patients with and without DKD (2016–2019)

Variables	DKD	No DKD	*P*
**N**	9583	392,739	-
**Total comorbidity rate, %**	2.38%		
**Age in years, mean (quartile)**	74	67	< 0.001
**Age distribution, n (%)**			< 0.001
18–44	48 (0.50%)	13,352 (3.40%)	
45–64	1621 (16.92%)	144,963 (36.91%)	
65–74	3199 (33.38%)	130,042 (33.11%)	
≥ 75	4711 (49.16%)	104,382 (26.58%)	
**Gender, n (%)**			< 0.001
Male	4801 (50.10%)	168,010 (42.78%)	
Female	4782 (49.90%)	224,685 (57.22%)	
**Race, n (%)**			< 0.001
White	7269 (75.85%)	325,349 (82.84%)	
Black	1208 (12.61%)	27,776 (7.07%)	
Hispanic	447 (4.66%)	14,189 (3.61%)	
Asian or Pacific Islander	153 (1.60%)	3680 (0.94%)	
Native American	55 (0.57%)	1191 (0.30%)	
Other	451 (4.71%)	20,554 (5.23%)	
**Insurance, n (%)**			< 0.001
Medicare	7753 (80.90%)	228,162 (58.10%)	
Medicaid	277 (2.89%)	19,113 (4.87%)	
Private insurance	1352 (14.11%)	132,981 (33.86%)	
Self-pay	46 (0.48%)	2908 (0.74%)	
No charge	4 (0.04%)	220 (0.06%)	
Other	151 (1.58%)	9355 (2.38%)	
**Elixhauser score, n (%)**			< 0.001
< 0	1027 (10.72%)	270,103 (68.77%)	
0–3	1366 (14.25%)	40,474 (10.31%)	
> 3	7190 (75.03%)	82,162 (20.92%)	
**Renal funtion, n (%)**			< 0.001
ESRD	649 (6.78%)	1032 (0.26%)	
Non-ESRD	8927 (93.22%)	391,714 (99.74%)	

### Patient demographics after propensity score matching

This study utilized the Elixhauser Comorbidity Index (ECI), a validated tool for quantifying the burden of comorbidities and their potential impact on short-term mortality risk, to assess patient health status [14]. To control for confounding demographic factors such as age, gender, race, and ECI score, a 1:1 propensity score matching (PSM) approach was implemented [13] (Table 2).

**Table 2:** Demographics of DKD and the matched cohort of total hip arthroplasty patient (2016–2019)

Variables	DKD	Matched controlled	*P*
**N**	9578	9578	
**Age distribution, n(%)**			0.563
18–44	48 (0.50%)	59 (0.62%)	
45–64	1621 (16.92%)	1666 (17.39%)	
65–74	3199 (33.40%)	3156 (32.95%)	
≥ 75	4711 (49.19%)	4697 (49.04%)	
**Gender, n(%)**			0.806
Male	4796 (50.07%)	4779 (49.90%)	
Female	4782 (49.93%)	4799 (50.10%)	
**Race, n(%)**			0.812
White	7269 (75.89%)	7240 (75.59%)	
Black	1204 (12.57%)	1209 (12.62%)	
Hispanic	447 (4.67%)	429 (4.48%)	
Asian or Pacific Islander	152 (1.59%)	172 (1.80%)	
Native American	55 (0.57%)	61 (0.64%)	
Other	451 (4.71%)	467 (4.88%)	
**Insurance, n(%)**			0.934
Medicare	7752 (80.94%)	7763 (81.05%)	
Medicaid	277 (2.89%)	295 (3.08%)	
Private insurance	1348 (14.07%)	1327 (13.85%)	
Self-pay	46 (0.48%)	40 (0.41%)	
No charge	4 (0.04%)	3 (0.03%)	
Other	151 (1.58%)	150 (1.57%)	
**Elixhauser score, n%**			0.320
< 0	1027 (10.72%)	979 (10.22%)	
0–3	1366 (14.26%)	1422 (14.85%)	
> 3	7185 (75.02%)	7177 (74.93%)	

### Data analysis

All data were statistically analyzed using IBM Statistical Package for the Social Sciences (SPSS) Statistics 25.0. A comparison of surgical complications, postoperative complications, mortality, LOS, total hospital costs and renal function between patients with DKD and their matched non-DKD controls was conducted. To control for potential confounding factors while comparing surgical and perioperative complications, mortality, LOS, and total hospital costs, we employed 1:1 propensity score matching based on age, gender, race, Elixhauser Comorbidity Index, and insurance type. Subsequently, a multivariate logistic regression analysis was implemented to estimate the advantage ratio (AR) for surgical and postoperative complications during the as-needed period in DKD patients relative to the matched non-DKD cohort.

## Results

### DKD incidence in THA patients from 2016 to 2019

Analysis of the National Inpatient Sample database revealed a steadily increasing prevalence of DKD among total hip arthroplasty patients from 2016 to 2019. Annual DKD incidence escalated from 1.13% to 3.07%, with a slight deceleration in 2019 compared to previous years. The cumulative four-year incidence of DKD following THA was 2.38%.

### Characteristics of DKD patients

DKD patients exhibited a significantly older age profile compared to non-DKD counterparts (mean age 74 vs. 67 years, *P* < 0.0001). Furthermore, DKD was associated with a marked elevation in ECI scores, with over 75% of DKD patients exhibiting scores exceeding 3 compared to only 20.92% in the non-DKD group. The proportion of patients with ESRD in the DKD population is more than ten times higher than in the non-DKD population (6.78% vs. 0.26%, *P* < 0.0001).

### Perioperative surgical and medical complications

**Fig. 2:**
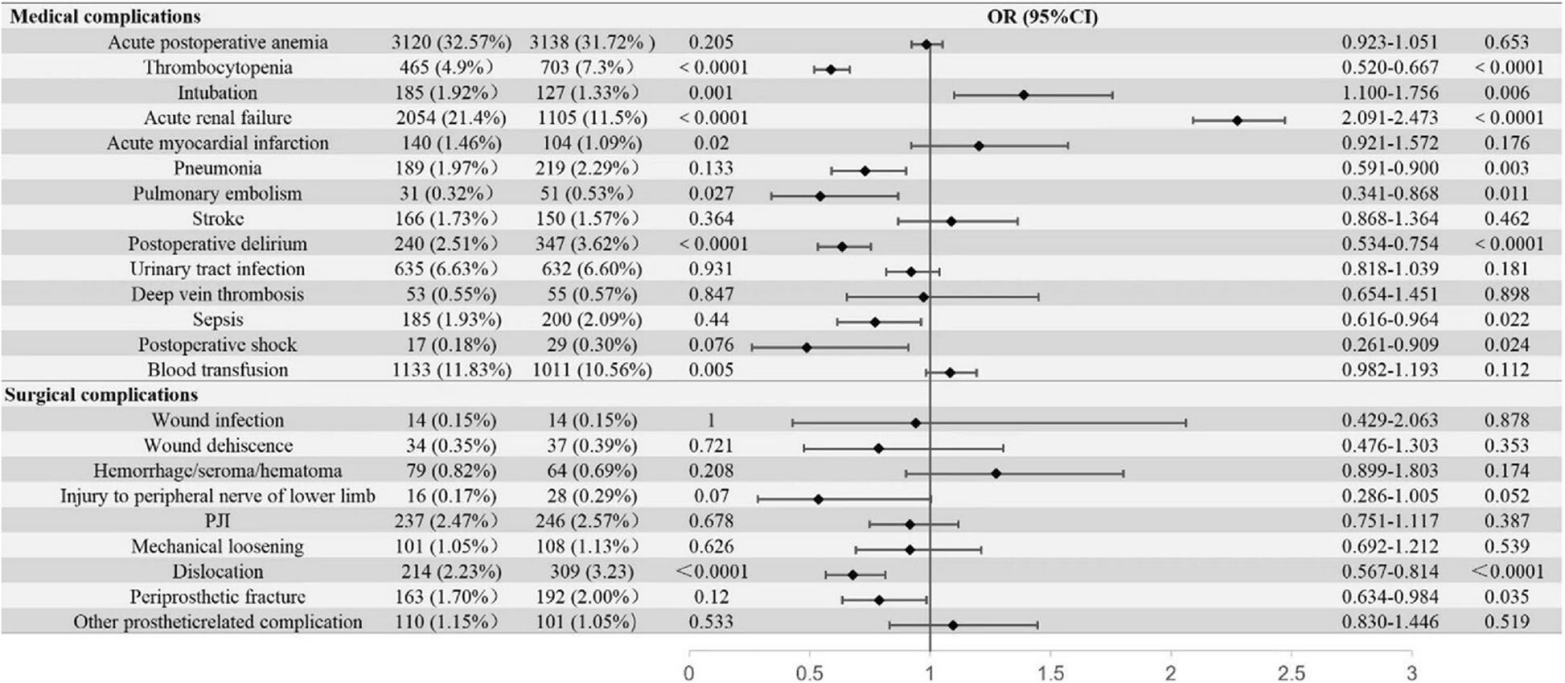
Univariate analysis & multivariate logistic regression analysis of medical complications and surgical complications in patients undergoing total hip arthroplasty with and without DKD

In terms of medical complications, DKD patients exhibited a significantly higher risk of acute renal failure compared to the matched cohort (OR 2.71 95% CI 2.491–2.952; *P* < 0.0001) (Fig. 2). Conversely, in terms of Thrombocytopenia, compared with the matched group, patients with DKD have a lower risk after THA (OR 0.759; 95% CI 0.668–0.862; *P* < 0.001) (Fig. 2).

### Postoperative outcomes in patients with DKD

Neither LOS nor total hospital cost demonstrated a statistically significant difference between DKD and non-DKD patients in the propensity-matched cohort. Average LOS remained comparable at 4.22 and 4.21 days, respectively (*P* = 0.109). Likewise, the mean total hospital cost for DKD ($81,632.31) did not differ significantly from non-DKD patients ($82,024.61) (*P* = 0.087) (Table 3).

**Table 3:** Hospital outcomes for patients undergoing total hip arthroplasty with and without DKD

Parameter	DKD	Matched controls	*P* value
*Length of stay (days)*			
Mean (SD)	4.22 (4.335)	4.21 (5.158)	0.109
Percent difference (95%CI)	4.11 to 4.32	4.13 to 4.30	0.109
*Charges (S)*			
Mean (SD)	81,632.31 (68,363.782)	82,024.61 (67,360.049)	0.087
Percent difference (95%CI)	80,675.44 to 83,373.79	80,263.04 to 83,001.59	0.087
*Mortality rate (%)*			
Rate	1.01	0.86	0.292
Percent difference (95%CI)	0.010023 (0.012226 to 0.002126)	0.008561 (0.010616 to 0.006815)	

*OR* Odds ratio, *CI* Confidence interval

## Discussion

This study investigated the potential association between DKD and an elevated risk of THA complications. Our findings suggest that DKD, may be a significant independent risk factor for short-term complication rates in THA recipients [15]. Despite the presence of DKD in 2.38% of total hip arthroplasty recipients in our study, no statistically significant increase in length of stay was observed compared to patients without DKD. Interestingly, this finding contrasts with the general expectation of increased healthcare utilization in patients with chronic comorbidities. This may be partially explained by the inherent selection bias of patients undergoing orthopedic surgery, where pre-existing bone fragility associated with DKD, potentially arising from cellular abnormalities, matrix interactions, immune and vascular changes, and musculoskeletal maladaptation to chronic hyperglycemia, might conversely promote earlier intervention for hip osteoarthritis [5, 16, 17]. Current evidence suggests that the risk of suffering from orthopedic conditions such as fractures [6] and secondary osteoporosis [7] is increased significantly in diabetes patients. Our study elucidates that the elevated risk can be attributed to the additional care required for patients with DKD.

Our investigation revealed an association between AKI and DKD. Among the 2054 DKD patients included in our study who underwent THA, 21.4% experienced AKI. In contrast, only 1.5% of the general THA patients developed AKI during their index hospitalization for THA [18]. The present study corroborates past findings by demonstrating a heightened risk of AKI in DM patients undergoing THA, use of specific drugs, presence of sepsis/septic shock, and even those lacking overt triggering factors [19, 20]. Nitric oxide (NO), a key endothelium-derived vasodilator, exhibits diminished production in DKD due to reduced activity of endothelial nitric oxide synthase (eNOS) [20, 21]. Genetic variations impacting NO synthesis have been linked to facilitate the exacerbation of diabetic nephropathy [22]. Collectively, these disruptions in NO metabolism sensitize the renal vasculature to vasoconstrictive stimuli [23]. Consequently, DM subjects are at increased risk of factors compromising oxygen transport to renal tubules, compounded by the enhanced oxygen demands imposed on these tubules by hyperfiltration, a hallmark of diabetic nephropathy [24]. Studies have indicated that as diabetes progresses, leading to a gradual decline in proteinuria and glomerular filtration rate (GFR) over the long term, individuals with DKD are more prone to experiencing AKI compared to those without DKD [25].

Apart from AKI, some medical complications were also significant (*P* ≤ 0.05) between the DKD and the matched group. Compared to previous study, the incidence of postoperative thrombocytopenia (4.9% vs. 0.37%), pulmonary embolism (0.32% vs. 0.2%), acute myocardial infarction (1.46% vs. 0.51%) and blood transfusion (11.83% vs. 0.7%) in patients with DKD receiving THA were higher, which may be related to decreased renal function/erythropoietin production, iron deficiency, side effects of hypoglycaemic drugs and diabetic microangiopathy in patients with DKD [26, 27]. A previous retrospective study showed that DKD is an independent risk factor for DVT during the perioperative period of THA [28]. Therefore, we suspect that patients with DKD are more likely to have deep vein thrombosis, pulmonary embolism, and other embolic events during the perioperative period of THA than patients without DKD. Interestingly, we observed a similar odds ratio for deep vein thrombosis, while a significant risk difference was observed in the ratio between the DKD group with positive pulmonary thrombosis and the matched group (0.32% vs. 0.53%). This may be due to the following reasons: 1 Some patients with advanced DKD often take anticoagulant drugs or antiplatelet drugs to reduce the occurrence of cardiovascular and cerebrovascular events [29]. 2 Doctors may perform more thorough assessments and preparations for patients with diabetes before surgery This may include more frequent blood tests, electrocardiograms, and other tests to ensure the safety of patients during surgery and to implement necessary preventive measures to reduce the risk of complications. In contrast, complications such as postoperative anemia, urinary tract infection, deep vein thrombosis, and sepsis did not show any significant differences between the DKD and matched groups, however, previous studies have reported significant differences, which may be attributed to consistencies in baseline data between the two groups after PSM [30]. The study identified wound infection, wound dehiscence and so on as a potential complication, but it did not reach statistical significance. The authors suggest that this may be attributed to the provision of adequate nursing care, nutritional support, and medication adherence, as reported in previous studies [10].

AKI has been established as a major complication associated with major surgery, with orthopedic procedures (including THA) identified as a high-risk category [18], highlighted by a pivotal study analyzing data from 161,185 Veterans Health Administration patients revealing a relative risk of 0.70 for developing AKI postoperatively in orthopedic surgery (95% CI: 0.67–0.73) [31]. This finding complements prior evidence suggesting a higher-than-anticipated prevalence of AKI in the perioperative setting [32]. The underlying mechanisms of perioperative AKI often involve hypoperfusion and inflammation [33, 34]. Perioperative hypovolemia, vasodilatory anesthetic effects, and cardio-inhibition can contribute to reduced blood pressure and subsequent renal hypoperfusion [35]. While the kidneys possess remarkable autoregulatory capacity to maintain perfusion, nonsteroidal anti-inflammatory drugs (NSAIDs) commonly used perioperatively can further compromise renal blood flow and GFR by inhibiting cyclooxygenase and prostaglandin synthesis [36].

Given the potential adverse impact of DKD on THA outcomes, for orthopaedic surgeons, during the perioperative period of THA, the following points need to be noted 1. Extensive laboratory evaluation of DKD severity and metabolic control. Targeted optimization of the laboratory indicators of DKD, including blood sugar levels, creatinine and urinary protein etc. 2. Comprehensive assessment of renal function through a battery of appropriate diagnostic tests. Preoperative interventions should be administered to enhance renal function, thereby mitigating the incidence of complications, particularly AKI, following hip arthroplasty. Attention should also be paid to the balance of patient's inflow and outflow to reduce the incidence of renal hypoperfusion [32]. 3. Antibiotics should be selected carefully, prioritizing those with low renal toxicity. Dosage should be adjusted based on the patient's preoperative renal function. Additionally, attention should be paid to the duration of antibiotic treatment [34]. 4. If there are signs of severe infection, such as fever and septic shock, during the perioperative period, prompt action is necessary. To evaluate the systemic inflammation of patients, surgeons should measure cytokines such as IL-6 and IL-24, as well as procalcitonin and C-reactive protein. This can assist surgeons in selecting appropriate anti-inflammatory medication [34, 35].

Our study, like other large database analyses, is susceptible to inherent limitations due to potential coding differences and data entry errors [37], which could potentially lead to an underestimation of post-THA complication rates. Additionally, the available variables and ICD-10 codes in the National Inpatient Sample database did not encompass key metrics like urine microalbumin/creatinine ratio, creatinine levels, or 24-h urine total protein quantification, hindering our ability to assess kidney function and preoperative kidney management. Meanwhile, in previous studies, we have found that DKD patients undergoing renal transplant have a lower incidence of postoperative complications compared to DKD patients undergoing dialysis. Unfortunately, in our study, we were unable to further compare the two due to the impact of missing data values [38]. Therefore, further prospective studies are crucial to investigate the impact of kidney function management on postoperative outcomes. Furthermore, this research focuses on the impact of DKD on the perioperative period of THA, as DKD is the main cause of chronic kidney disease. Other renal diseases' potential impact on THA was not addressed in this article and requires further research. Besides, the absence of a defined timeframe (e.g., 30 or 90 days) to differentiate between early and late complications presents another limitation [39]. While our study focused only on early complications, this prevents a comprehensive evaluation of long-term consequences like cup or stem loosening.

## Conclusions

This study demonstrates that DKD is a significant risk factor for AKI in patients undergoing total hip arthroplasty. Optimizing preoperative kidney function through appropriate interventions might decrease the risk of poor prognosis in this population. More prospective research is warranted to investigate the potential of targeted kidney function improvement strategies in reducing AKI rates after THA. The findings of this study hold promise for enhancing preoperative counseling by surgeons, enabling them to provide DKD patients undergoing THA with more precise information regarding the risks associated with their condition.

## Data Availability

The data that support the findings of this study are available from the corresponding author, MC, upon reasonable request.

## Abbreviations

DKD: Diabetic kidney Disease
THA: Total hip arthroplasty
LOS: Length of stays
PSM: Propensity score matching
ECI: Elixhauser Comorbidity Index
ESRD: End-stage renal disease
